# Crystal structures of 2-amino-4,4,7,7-tetra­methyl-4,5,6,7-tetra­hydro-1,3-benzo­thia­zol-3-ium benzoate and 2-amino-4,4,7,7-tetra­methyl-4,5,6,7-tetra­hydro-1,3-benzo­thia­zol-3-ium picrate

**DOI:** 10.1107/S2056989017011446

**Published:** 2017-08-08

**Authors:** Belakavadi K. Sagar, Marisiddaiah Girisha, Hemmige S. Yathirajan, Ravindranath S. Rathore, Christopher Glidewell

**Affiliations:** a Department of Studies in Chemistry, University of Mysore, Manasagangotri, Mysuru 570 006, India; b Centre for Biological Sciences (Bioinformatics), School of Earth, Biological and Environmental Sciences, Central University of South Bihar, Patna 800 014, India; c School of Chemistry, University of St Andrews, St Andrews, Fife KY16 9ST, Scotland

**Keywords:** crystal structures, mol­ecular conformation, conformational disorder, charge delocalization, hydrogen bonding

## Abstract

In both 2-amino-4,4,7,7-tetra­methyl-4,5,6,7-tetra­hydro-1,3- benzo­thia­zol-3-ium benzoate, C_11_H_19_N_2_S^+^·C_7_H_5_O_2_^−^, (I), and 2-amino-4,4,7,7-tetra­methyl-4,5,6,7-tetra­hydro-1,3-benzo­thia­zol-3-ium picrate (2,4,6-tri­nitro­phenolate), C_11_H_19_N_2_S^+^·C_6_H_2_N_3_O_7_^−^, (II), the cations are conformationally chiral as the six-membered rings adopt half-chair conformations, which are disordered over two sets of atomic sites giving approximately enanti­omeric disorder. For both cations, the bond lengths indicate delocalization of the positive charge comparable to that in an amidinium cation. The bond lengths in the picrate anion in (II) are consistent with extensive delocalization of the negative charge into the ring and onto the nitro groups, in two of which the O atoms are disordered over two sets of sites. In (I), the ionic components are linked by N—H⋯O hydrogen bonds to form a chain of rings, and in (II), the N—H⋯O hydrogen bonds link the components into centrosymmetric four-ion aggregates containing seven hydrogen bonded rings of four different types.

## Abstract 2

In each of the title compounds, the cation is conformationally chiral, exhibiting conformational disorder, while two of the nitro groups in the picrate anion also exhibit disorder. In the benzoate salt, the ions are linked into a chain of rings by N—H⋯O hydrogen bonds, whole in the picrate salt, the hydrogen-bonded four-ion aggregate contains four different types of ring.

## Chemical context   

Benzo­thia­zoles are an important class of heterocyclic compounds which possess a wide spectrum of biological properties, including analgesic, anti­convulsant, anti­helmintic, anti-inflammatory anti­malarial, anti­microbial, anti­tubercular, and anti­tumour, activity, as well as anti­oxidant and fungicidal activity (Imramovský *et al.*, 2013 ▸; Smita Revankar *et al.*, 2014 ▸; Naga Raju *et al.*, 2015 ▸; Ranga *et al.*, 2015 ▸). In addition, substituted 2-amino­thia­zole derivatives are important as potent and selective human adenosine A3 receptor antagonists (Jung *et al.*, 2004 ▸). Prompted by the importance of benzo­thia­zoles in general, we have now determined the mol­ecular and supra­molecular structures of two salts derived from a substituted benzo­thia­zole, 2-amino-4,4,7,7-tetra­methyl-4,5,6,7-tetra­hydro-1,3-benzo­thia­zole, namely 2-amino-4,4,7,7-tetra­methyl-4,5,6,7-tetra­hydro-1,3-benzo­thia­zol-3-ium benz­oate (I) and 2-amino-4,4,7,7- tetra­methyl-4,5,6,7-tetra­hydro-1,3-benzo­thia­zol-3-ium picrate (2,4,6-tri­nitro­phenolate) (II), which we report here. The compounds were prepared by acid–base reactions between the neutral benzo­thia­zole and the appropriate acid in methano­lic solution.
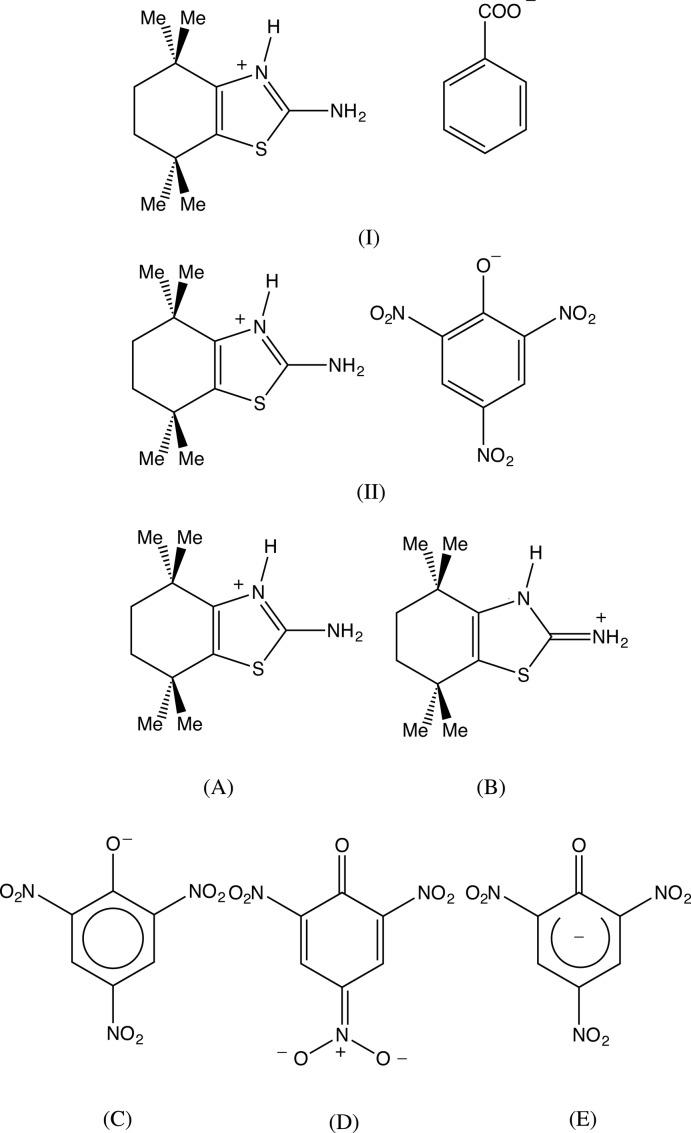


## Structural commentary   

**Figure 1:**
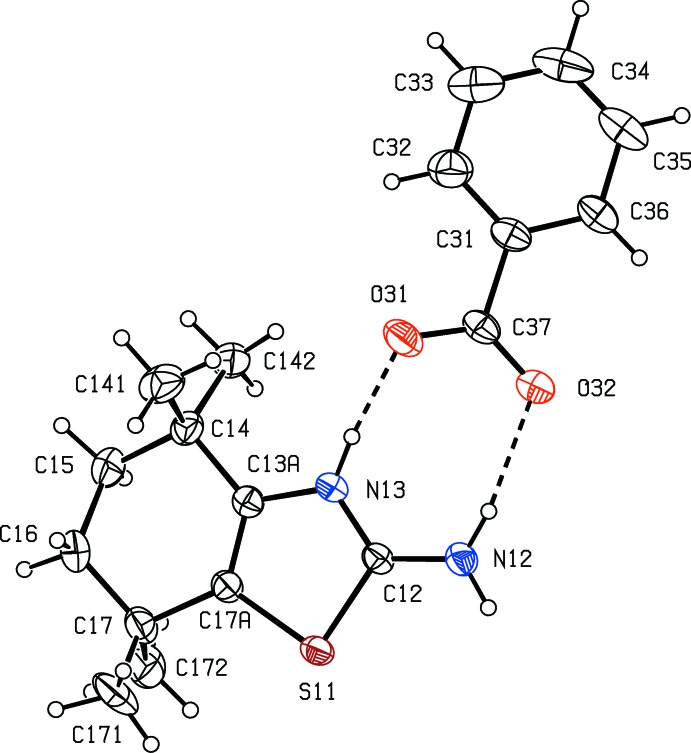
The independent ionic components of compound (I), showing the atom-labelling scheme. Displacement ellipsoids are drawn at the 30% probability level, and the two N—H⋯O hydrogen bonds within the selected asymmetric unit are shown as dashed lines.

**Figure 2:**
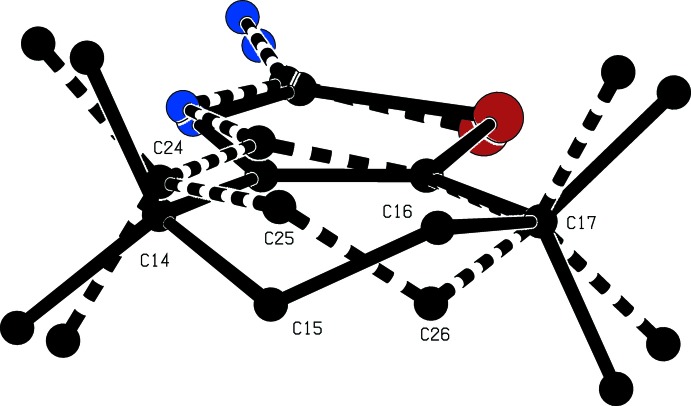
The disordered cation in compound (I), showing the approximately enanti­omorphic nature of the two disorder components. For the sake of clarity the H atoms and most of the atom labels have been omitted: the major form is drawn as solid lines and the minor form as broken lines.

Compound (I) consists of a reduced benzo­thia­zolium cation in which protonation has occurred exclusively at atom N13, and a benzoate anion and the two ions within the selected asymmetric unit are linked by two fairly short and nearly linear N—H⋯O hydrogen bonds, forming an 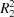
(8) motif (Fig. 1 ▸ and Table 1 ▸). In the cation, the six-membered ring is disordered over two sets of atomic sites with occupancies 0.721 (5) and 0.279 (5), and each disorder component adopts a half-chair conformation (Fig. 2 ▸). The ring-puckering parameters calculated for the atom sequence (C*x*3*A*,C*x*4,C*x*5,C*x*6,C*x*7,C*x*7*A*), where *x* = 1 for the major conformer and *x* = 2 for the minor form, of *Q* = 0.452 (5) Å, θ = 47.3 (8)° and φ = 146.1 (10)° when *x* = 1, with corresponding values *Q* = 0.453 (13) Å, θ = 138.5 (19)° and φ = 340 (3)° when *x* = 2. For an idealized half-chair form the puckering angles are θ = 50.8° and φ = (60*k* + 30)°, where *k* represents an integer. In each of (I) and (II), in fact, the cation exhibits no inter­nal symmetry and hence is conformationally chiral: in each case the space group confirms the presence of equal numbers of the two conformational enanti­omers. In the benzoate anion in (I), the carboxyl group makes a dihedral angle of 10.5 (2)° with the aryl ring, and the two C—O distances are identical within experimental uncertainty, 1.252 (3) and 1.255 (3) Å, consistent with the complete transfer of a proton from the acid component to atom N13, as deduced from difference maps and confirmed by the refinement.

**Figure 3:**
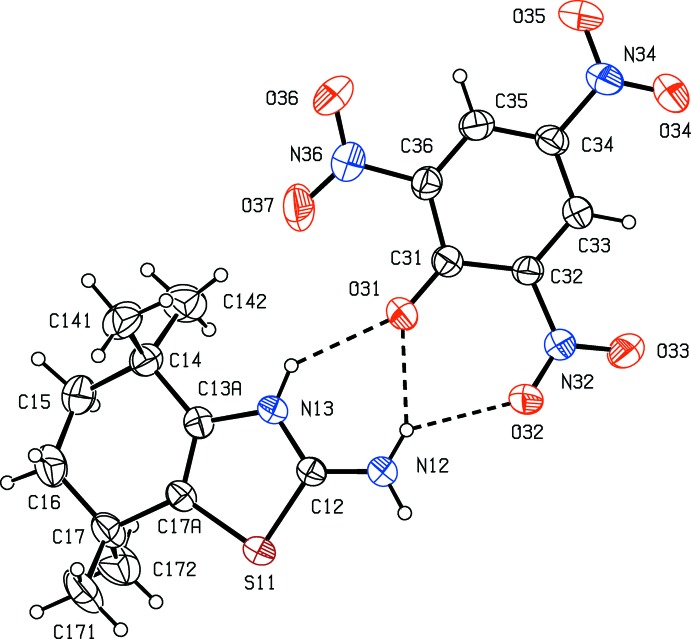
The independent ionic components of compound (II), showing the atom-labelling scheme. For the sake of clarity, only the major disorder components are shown. Displacement ellipsoids are drawn at the 30% probability level, and the N—H⋯O hydrogen bonds within the selected asymmetric unit are shown as dashed lines.

Compound (II) contains the same cation as (I) along with a picrate (2,4,6-tri­nitro­phenolate) anion, and the two ions in the selected asymmetric unit are linked by a two-centre N—H⋯O hydrogen bond and a three-centre N—H⋯(O)_2_ hydrogen bond, generating two edge-fused rings of 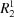
(6) and 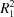
(6) types (Fig. 3 ▸ and Table 2 ▸).

The cation again exhibits conformational disorder over two sets of atomic sites having occupancies 0.575 (4) and 0.425 (4). For the major conformer, the ring-puckering parameters, calculated for the atom sequence (C*x*3*A*,C*x*4,C*x*5,C*x*6,C*x*7,C*x*7*A*) are *Q* = 0.444 (10) Å, θ = 41.9 (15)° and φ = 150 (2) when *x* = 1 and *Q* = 0.441 (14) Å, θ = 136 (2)° and φ = 328 (3)° when *x* = 2, so that the ring- puckering parameters are very similar to those found in compound (I). Thus in each compound the puckering amplitude for the two conformers are very similar, and the puckering angles, related approximately by θ_min_ = (180 − θ_maj_) and φ_min_ = (180 + φ_maj_), where min and maj refer to the minor and major components, indicate clearly the approximately enanti­omorphic relationship between the two conformers (Fig. 2 ▸).

In both compounds the bond distances C12—N12 and C12—N13 are nearly identical, 1.329 (6) and 1.323 (3) Å respectively in (I) and 1.312 (3) and 1.336 (9) Å in (II), indicative of significant delocalization of the positive charge into the amino group with significant contributions to the electronic structure from the forms (A) and (B), comparable to an amidinium cation (see Scheme). This explains not only why the site of protonation is exclusively at the ring N atom, since protonation of the amino group would not permit any charge delocalization, but also the observation that the amino N atom does not act as a hydrogen-bond acceptor.

**Figure 4:**
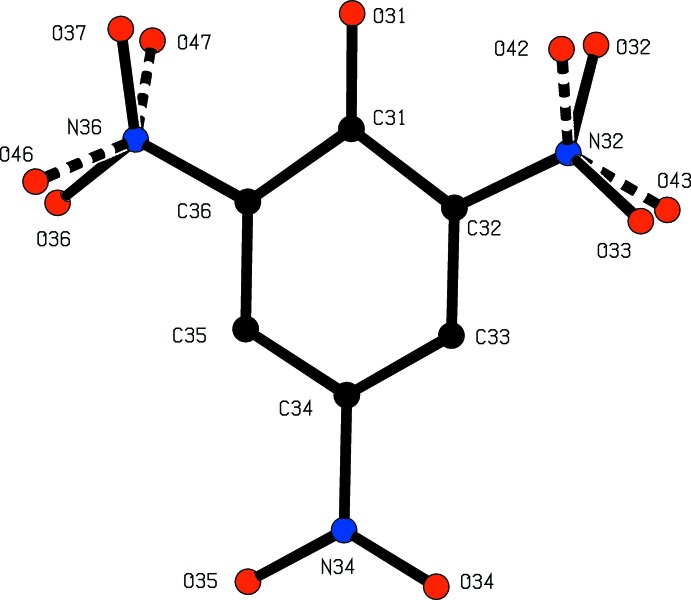
The disordered anion in compound (II), showing the two orientations of two of the nitro groups: for the sake of clarity the H atoms have been omitted,

In the picrate anion of (II), two of the three independent nitro groups adopt two different orientations and the occupancies for the two orientations bonded to atoms C32 and C36 are 0.769 (7) and 0.231 (7), and 0.789 (6) and 0.211 (6) respectively (Fig. 4 ▸). The major and minor conformations at C32 make dihedral angles of 17.9 (3) and 27.2 (7)° with the ring, with an angle of 44.9 (7)° between the two orientations, and the corresponding values for the nitro group at C36 are 12.0 (2), 39.0 (8) and 50.4 (8)°. By contrast, the fully ordered nitro group at C34 makes a dihedral angle of only 4.5 (2)° with the ring. The C—O distance, 1.241 (3), is short for its type [mean value (Allen *et al.*, 1987 ▸) 1.362 Å, lower quartile value 1.353 Å], and the C—N distances, range 1.442 (3)–1.458 (3) Å, are all somewhat short for their type (mean value 1.468 Å, lower quartile value 1.460 Å): in addition, the bonds C31—C32 and C31—C36 are significantly longer than the other C—C distances in this ring. These observation, taken together, indicate that the quinonoid form (D), and its *o*-quinonoid isomers, and the ketonic form (E) are significant contributors to the overall electronic structure of the anion in addition to the classically delocalized form (C) (see Scheme).

## Supra­molecular inter­actions   

**Figure 5:**
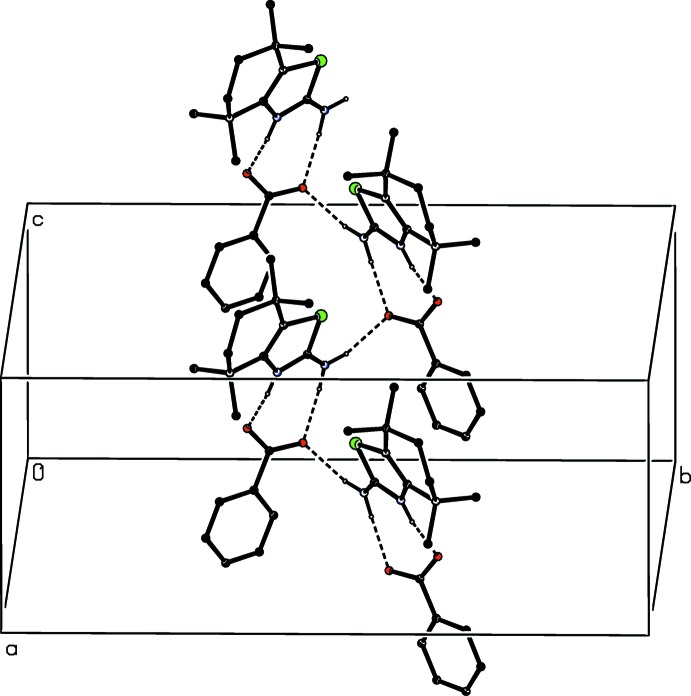
Part of the crystal structure of compound (I) showing the formation of a chain of rings running parallel to [001]. Hydrogen bonds are shown as dashed lines and for the sake of clarity the H atoms bonded to C atoms have been omitted.

The major and minor conformers of the cation in (I) and those of both ions in (II) are involved in very similar patterns of hydrogen bonding (Tables 1 ▸ and 2 ▸), so that it is necessary to discuss only those formed by the major conformers. Because of the charge delocalization in both ions in each of (I) and (II), as noted above, all of the N—H⋯O inter­actions in both compounds can be regarded as charge-assisted hydrogen bonds (Gilli *et al.*, 1994 ▸). In addition to the two N—H⋯O hydrogen bonds within the selected asymmetric unit of compound (I) (Fig. 1 ▸), the structure contains a third such inter­action which links the cation-anion pairs which are related by the *c*-glide plane at *y* = 0.5 into a 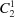
(4) 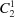
(8)[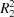
(8)] chain of rings running parallel to the [001] direction (Fig. 5 ▸).

**Figure 6:**
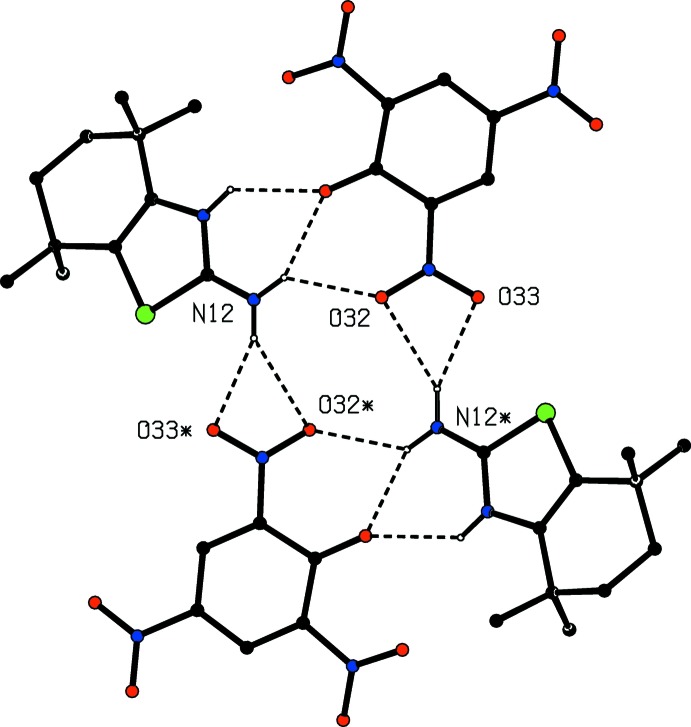
Part of the crystal structure of compound (II) showing the formation of a centrosymmetric four-ion aggregate. For the sake of clarity, only the major disorder components are shown, and the H atoms bonded to C atoms and the unit cell outline have been omitted. The atoms marked with an asterisk (*) are at the symmetry position (1 − *x*, −*y*, 1 − *z*).

In addition, the N—H⋯O hydrogen bonds within the selected asymmetric unit of (II) (Fig. 3 ▸), the structure contains one further three-centre N—H⋯(O)_2_ hydrogen bond, and the hydrogen bonds together generate a four-ion aggregate in which a centrosymmetric 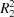
4(8) ring is surrounded by three inversion-related pairs of rings, one each of 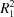
(4), 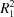
(6) and 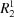
(6) types, so that, in total, there are seven hydrogen-bonded rings of four different types in the aggregate (Fig. 6 ▸). It is notable that only one of the nitro groups in (II) participates in the hydrogen bonding, and that both C—H⋯π(arene) and aromatic π–π stacking inter­actions are absent from both structures.

## Database survey   

It is of inter­est briefly to survey the structures of some related amino-substituted benzo-1,3-thia­zoles. In the structure of 2-amino-6-nitro­benzo-1,3-thia­zole (Glidewell *et al.*, 2001 ▸), a combination of N—H⋯N and N—H⋯O hydrogen bonds generates a three-dimensional framework structure, while the monohydrate of the same benzo­thia­zole, also forms a three-dimensional framework structure, but now built from a combination of N—H⋯N, N—H⋯O and O—H⋯O hydrogen bonds (Lynch, 2002 ▸): in neither of these structures does the amino N atom act as a hydrogen-bond acceptor, just as found here in the structures of (I) and (II). We note also that in *trans*-bis­(2-amino-6-nitro­benzo-1,3-thia­zole)di­chloro­platinum(II), which crystallizes as a tetra­kis­(di­methyl­formamide) solvate (Lynch & Duckhouse, 2001 ▸), the benzothiazole ligand coordinates to the metal centre *via* the ring N atom, rather than *via* the amino N atom. Finally in 2-amino-6-nitro­benzo-1.3-thia­zol-3-ium hydrogen sulfate (Qian & Huang, 2011 ▸), the protonation of the benzo­thia­zole component occurs exclusively at the ring N atoms and the ions are linked by a combination of N—H⋯O and O—H⋯O hydrogen bonds to form a sheet structure, again with the amino group acting as a double donor of hydrogen bonds, but not as an acceptor.

## Synthesis and crystallization   

2-Amino-4,4,7,7-tetra­methyl-4,5,6,7-tetra­hydro-1,3-benzo­thia­zole (200 mg, 0.94 mmol) and the equivalent amount of the respective acid *i.e.* benzoic acid (119.4 mg, 0.94 mmol) for (I) and picric acid (229 mg, 0.94 mmol) for (II) were dissolved together in hot methanol. The resulting solutions were allowed to cool slowly to ambient temperature, and the crystalline products were collected by filtration and dried in air. Crystals suitable for single-crystal X-ray diffraction were selected directly from the samples as prepared; m.p. (I) 457 K, (II) 483 K.

## Refinement   

Crystal data, data collection and structure refinement details are summarized in Table 3 ▸. It was apparent from an early stage in the refinements that in both (I) and (II) the cation was disordered over two sets of atomic sights corresponding to two different conformations of the six-membered ring, and that two of the nitro groups in the anion of (II) were disordered, again over two sets of atomic sites corresponding to different orientations relative to the aryl ring. For the minor conformers of the cations, the bonded distances and the one-angle non-bonded distances were restrained to be the same as the corresponding distances in the major conformer, subject to s.u.s of 0.005 and 0.01 Å, respectively; similar restraints were applied to the minor conformations of the disordered nitro groups in the anion of (II). In addition, the anisotropic displacement parameters for pairs of atoms occupying essentially the same physical space were constrained to be identical. Subject to these conditions, the occupancies of the two cation conformations in (I) refined to 0.721 (5) and 0.279 (5), and those in (II) refined to 0.575 (4) and 0.425 (4), while those of the nitro groups in (II) bonded to C32 and C36 refined to 0.769 (7) and 0.231 (7), and 0.789 (6) and 0.211 (6) respectively.

All H were treated as riding atoms in geometrically idealized positions with distances C—H = 0.93 Å (aromatic), 0.96 Å (CH_3_) or 0.97 Å (CH_2_) and N—H = 0.86 Å, and with *U*_iso_(H) = *kU*_eq_ (C), where *k* = 1.5 for the methyl groups which were permitted to rotate but not to tilt and 1.2 for all other H atoms. One bad outlier reflection (39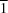) was omitted from the final refinement of (I).

The correct orientation of the structure of (I), relative to the polar axis direction, was established by means of the Flack *x* parameter (Flack, 1983 ▸), calculated (Parsons *et al.*, 2013 ▸) using 1373 quotients of the type [(*I* ^+^)−(*I* ^−^)]/[(*I* ^+^)+(*I* ^−^)], and by means of the Hooft *y* parameter (Hooft *et al.*, 2010 ▸): *x* = 0.061 (7) and *y* = 0.0561 (8): use of the TWIN/BASF procedure in *SHELXL* for the determination of the Flack *x* parameter gave a less well defined value, *x* = 0.053 (18). In the final analysis of variance for compound (II), there was a large value, 6.892, of K = [mean(*F_o_* ^2^)/mean(*F*_c_^2^)] for the group of 433 very weak reflections having *F*_c_/*F*_c_(max) in the range 0 < *F*_c_/*F*_c_(max) < 0.006.

## Supplementary Material

Crystal structure: contains datablock(s) global, I, II. DOI: 10.1107/S2056989017011446/lh5849sup1.cif

Structure factors: contains datablock(s) I. DOI: 10.1107/S2056989017011446/lh5849Isup2.hkl

Structure factors: contains datablock(s) II. DOI: 10.1107/S2056989017011446/lh5849IIsup3.hkl

Supporting information file. DOI: 10.1107/S2056989017011446/lh5849Isup4.cml

Supporting information file. DOI: 10.1107/S2056989017011446/lh5849IIsup5.cml

CCDC references: 1566446, 1566445

Additional supporting information:  crystallographic information; 3D view; checkCIF report

## Figures and Tables

**Table 1:** Hydrogen-bond geometry (Å, °) for (I)

*D*—H⋯*A*	*D*—H	H⋯*A*	*D*⋯*A*	*D*—H⋯*A*
N12—H12*A*⋯O32	0.86	2.10	2.918 (8)	158
N12—H12*B*⋯O32^i^	0.86	1.97	2.785 (9)	158
N13—H13⋯O31	0.86	1.77	2.621 (10)	174
N22—H22*A*⋯O32	0.86	2.13	2.86 (2)	142
N22—H22*B*⋯O32^i^	0.86	2.13	2.92 (2)	152
N23—H23⋯O31	0.86	1.74	2.56 (3)	157

Symmetry code: (i) 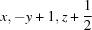.

**Table 2:** Hydrogen-bond geometry (Å, °) for (II)

*D*—H⋯*A*	*D*—H	H⋯*A*	*D*⋯*A*	*D*—H⋯*A*
N12—H12*A*⋯O32^i^	0.86	2.57	3.219 (10)	133
N12—H12*A*⋯O33^i^	0.86	2.31	3.039 (10)	142
N12—H12*A*⋯O42^i^	0.86	2.41	3.166 (16)	147
N12—H12*A*⋯O43^i^	0.86	2.58	3.197 (15)	129
N22—H22*A*⋯O32^i^	0.86	2.34	3.154 (14)	158
N22—H22*A*⋯O33^i^	0.86	2.40	3.143 (14)	146
N22—H22*A*⋯O42^i^	0.86	2.36	3.190 (19)	163
N22—H22*A*⋯O43^i^	0.86	2.46	3.197 (18)	144
N12—H12*B*⋯O31	0.86	2.11	2.855 (9)	145
N12—H12*B*⋯O32	0.86	2.20	2.870 (9)	134
N12—H12*B*⋯O42	0.86	2.30	2.932 (13)	131
N22—H22*B*⋯O31	0.86	1.97	2.704 (14)	142
N22—H22*B*⋯O32	0.86	2.14	2.768 (14)	130
N22—H22*B*⋯O42	0.86	2.16	2.730 (17)	123
N13—H13⋯O31	0.86	2.19	2.891 (14)	138
N23—H23⋯O31	0.86	2.15	2.81 (2)	134

Symmetry code: (i) 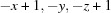.

**Table 3:** Experimental details

	(I)	(II)
Crystal data		
Chemical formula	C_11_H_19_N_2_S^+^·C_7_H_5_O_2_^−^	C_11_H_19_N_2_S^+^·C_6_H_2_N_3_O_7_^−^
*M*_r_	332.45	439.45
Crystal system, space group	Monoclinic, *C* *c*	Monoclinic, *P*2_1_/*n*
Temperature (K)	296	296
*a*, *b*, *c* (Å)	10.6089 (3), 22.7141 (5), 8.8959 (2)	10.7928 (2), 6.9591 (1), 28.0176 (5)
β (°)	122.211 (1)	97.408 (1)
*V* (Å^3^)	1813.73 (8)	2086.79 (6)
*Z*	4	4
Radiation type	Cu *K*α	Cu *K*α
μ (mm^−1^)	1.67	1.82
Crystal size (mm)	0.20 × 0.20 × 0.12	0.30 × 0.25 × 0.20

Data collection		
Diffractometer	Bruker Kappa APEXII	Bruker Kappa APEXII
Absorption correction	Multi-scan (*SADABS*; Bruker, 2012 ▸)	Multi-scan (*SADABS*; Bruker, 2012 ▸)
*T*_min_, *T*_max_	0.796, 0.819	0.696, 0.712
No. of measured, independent and observed [*I* > 2σ(*I*)] reflections	12953, 3209, 3138	40786, 4122, 3099
*R*_int_	0.028	0.060
(sin θ/λ)_max_ (Å^−1^)	0.619	0.618

Refinement		
*R*[*F* ^2^ > 2σ(*F* ^2^)], *wR*(*F* ^2^), *S*	0.028, 0.072, 1.05	0.053, 0.139, 1.07
No. of reflections	3209	4122
No. of parameters	260	336
No. of restraints	42	46
H-atom treatment	H-atom parameters constrained	H-atom parameters constrained
Δρ_max_, Δρ_min_ (e Å^−3^)	0.14, −0.10	0.25, −0.23
Absolute structure	Flack *x* determined using 1373 quotients [(*I* ^+^)−(*I* ^−^)]/[(*I* ^+^)+(*I* ^−^)] (Parsons *et al.*, 2013 ▸)	–
Absolute structure parameter	0.061 (7)	–

Computer programs: *APEX2* and *SAINT-Plus* (Bruker, 2012 ▸), *SHELXS86* (Sheldrick, 2008 ▸), *SHELXL2014* (Sheldrick, 2015 ▸) and *PLATON* (Spek, 2009 ▸).

## supplementary crystallographic information

### 2-Amino-4,4,7,7-tetramethyl-4,5,6,7-tetrahydro-1,3-benzothiazol-3-ium benzoate (I) Crystal data

**Table:**

C_11_H_19_N_2_S^+^·C_7_H_5_O_2_^−^	*F*(000) = 712
*M**_r_* = 332.45	*D*_x_ = 1.217 Mg m^−^^3^
Monoclinic, *C**c*	Cu *K*α radiation, λ = 1.54178 Å
*a* = 10.6089 (3) Å	Cell parameters from 3210 reflections
*b* = 22.7141 (5) Å	θ = 7.7–72.5°
*c* = 8.8959 (2) Å	µ = 1.67 mm^−^^1^
β = 122.211 (1)°	*T* = 296 K
*V* = 1813.73 (8) Å^3^	Block, colourless
*Z* = 4	0.20 × 0.20 × 0.12 mm

### 2-Amino-4,4,7,7-tetramethyl-4,5,6,7-tetrahydro-1,3-benzothiazol-3-ium benzoate (I) Data collection

**Table:**

Bruker Kappa APEXII diffractometer	3138 reflections with *I* > 2σ(*I*)
Radiation source: fine focus sealed tube	*R*_int_ = 0.028
φ and ω scans	θ_max_ = 72.5°, θ_min_ = 7.7°
Absorption correction: multi-scan (SADABS; Bruker, 2012)	*h* = −12→13
*T*_min_ = 0.796, *T*_max_ = 0.819	*k* = −28→28
12953 measured reflections	*l* = −10→11
3209 independent reflections	

### 2-Amino-4,4,7,7-tetramethyl-4,5,6,7-tetrahydro-1,3-benzothiazol-3-ium benzoate (I) Refinement

**Table:**

Refinement on *F*^2^	H-atom parameters constrained
Least-squares matrix: full	*w* = 1/[σ^2^(*F*_o_^2^) + (0.035*P*)^2^ + 0.3018*P*] where *P* = (*F*_o_^2^ + 2*F*_c_^2^)/3
*R*[*F*^2^ > 2σ(*F*^2^)] = 0.028	(Δ/σ)_max_ < 0.001
*wR*(*F*^2^) = 0.072	Δρ_max_ = 0.14 e Å^−^^3^
*S* = 1.05	Δρ_min_ = −0.10 e Å^−^^3^
3209 reflections	Extinction correction: SHELXL, Fc^*^=kFc[1+0.001xFc^2^λ^3^/sin(2θ)]^-1/4^
260 parameters	Extinction coefficient: 0.0076 (13)
42 restraints	Absolute structure: Flack *x* determined using 1373 quotients [(*I*^+^)-(*I*^-^)]/[(*I*^+^)+(*I*^-^)] (Parsons *et al.*, 2013)
Hydrogen site location: inferred from neighbouring sites	Absolute structure parameter: 0.061 (7)

### 2-Amino-4,4,7,7-tetramethyl-4,5,6,7-tetrahydro-1,3-benzothiazol-3-ium benzoate (I) Special details

**Table:**

Geometry. All esds (except the esd in the dihedral angle between two l.s. planes) are estimated using the full covariance matrix. The cell esds are taken into account individually in the estimation of esds in distances, angles and torsion angles; correlations between esds in cell parameters are only used when they are defined by crystal symmetry. An approximate (isotropic) treatment of cell esds is used for estimating esds involving l.s. planes.

### 2-Amino-4,4,7,7-tetramethyl-4,5,6,7-tetrahydro-1,3-benzothiazol-3-ium benzoate (I) Fractional atomic coordinates and isotropic or equivalent isotropic displacement parameters (Å^2^)

**Table:**

	*x*	*y*	*z*	*U*_iso_*/*U*_eq_	Occ. (<1)
S11	0.4896 (6)	0.4724 (3)	0.9010 (5)	0.0475 (7)	0.721 (5)
C12	0.397 (2)	0.4487 (11)	0.6831 (14)	0.0401 (6)	0.721 (5)
N13	0.4673 (17)	0.4048 (8)	0.6615 (8)	0.0420 (9)	0.721 (5)
H13	0.4313	0.3863	0.5626	0.050*	0.721 (5)
C13A	0.6050 (6)	0.3910 (3)	0.8127 (5)	0.0386 (11)	0.721 (5)
C14	0.7022 (5)	0.34297 (18)	0.8119 (5)	0.0483 (9)	0.721 (5)
C15	0.8542 (5)	0.3483 (2)	0.9885 (5)	0.0665 (11)	0.721 (5)
H15A	0.9108	0.3126	1.0059	0.080*	0.721 (5)
H15B	0.9089	0.3808	0.9791	0.080*	0.721 (5)
C16	0.8442 (6)	0.35787 (19)	1.1501 (5)	0.0700 (12)	0.721 (5)
H16A	0.9443	0.3586	1.2549	0.084*	0.721 (5)
H16B	0.7925	0.3247	1.1619	0.084*	0.721 (5)
C17	0.7639 (5)	0.4147 (2)	1.1447 (5)	0.0534 (6)	0.721 (5)
C17A	0.6315 (6)	0.4208 (3)	0.9579 (6)	0.0427 (5)	0.721 (5)
N12	0.2707 (7)	0.4723 (4)	0.5525 (10)	0.0471 (15)	0.721 (5)
H12A	0.2303	0.4590	0.4460	0.057*	0.721 (5)
H12B	0.2296	0.5009	0.5744	0.057*	0.721 (5)
C141	0.6274 (8)	0.2830 (2)	0.7895 (8)	0.0755 (16)	0.721 (5)
H14A	0.6909	0.2523	0.7921	0.113*	0.721 (5)
H14B	0.5338	0.2820	0.6778	0.113*	0.721 (5)
H14C	0.6108	0.2771	0.8845	0.113*	0.721 (5)
C142	0.7241 (7)	0.3516 (3)	0.6554 (6)	0.0628 (12)	0.721 (5)
H14D	0.7986	0.3246	0.6670	0.094*	0.721 (5)
H14E	0.7557	0.3912	0.6560	0.094*	0.721 (5)
H14F	0.6318	0.3442	0.5456	0.094*	0.721 (5)
C171	0.7103 (7)	0.4101 (3)	1.2740 (7)	0.0862 (18)	0.721 (5)
H17B	0.7913	0.3976	1.3882	0.129*	0.721 (5)
H17C	0.6307	0.3820	1.2295	0.129*	0.721 (5)
H17D	0.6753	0.4478	1.2850	0.129*	0.721 (5)
C172	0.8684 (5)	0.4675 (2)	1.1937 (7)	0.0728 (14)	0.721 (5)
H17E	0.9510	0.4634	1.3139	0.109*	0.721 (5)
H17F	0.8152	0.5031	1.1827	0.109*	0.721 (5)
H17G	0.9045	0.4692	1.1151	0.109*	0.721 (5)
S21	0.488 (2)	0.4759 (8)	0.882 (2)	0.0475 (7)	0.279 (5)
C22	0.392 (6)	0.447 (3)	0.670 (4)	0.0401 (6)	0.279 (5)
N23	0.468 (5)	0.405 (2)	0.652 (2)	0.0420 (9)	0.279 (5)
H23	0.4453	0.3908	0.5504	0.050*	0.279 (5)
C23A	0.5892 (17)	0.3843 (8)	0.8131 (15)	0.0386 (11)	0.279 (5)
C24	0.6860 (12)	0.3350 (5)	0.8173 (12)	0.0483 (9)	0.279 (5)
C25	0.8079 (12)	0.3238 (4)	1.0135 (12)	0.0665 (11)	0.279 (5)
H25A	0.7663	0.2998	1.0668	0.080*	0.279 (5)
H25B	0.8878	0.3015	1.0179	0.080*	0.279 (5)
C26	0.8729 (12)	0.3791 (4)	1.1235 (14)	0.0700 (12)	0.279 (5)
H26A	0.9173	0.4026	1.0725	0.084*	0.279 (5)
H26B	0.9517	0.3681	1.2426	0.084*	0.279 (5)
C27	0.7586 (12)	0.4170 (5)	1.1352 (14)	0.0534 (6)	0.279 (5)
C27A	0.6271 (16)	0.4222 (8)	0.9477 (16)	0.0427 (5)	0.279 (5)
N22	0.255 (2)	0.4633 (12)	0.544 (3)	0.0471 (15)	0.279 (5)
H22A	0.2099	0.4455	0.4433	0.057*	0.279 (5)
H22B	0.2111	0.4913	0.5639	0.057*	0.279 (5)
C241	0.590 (2)	0.2806 (7)	0.725 (2)	0.0755 (16)	0.279 (5)
H24A	0.6528	0.2475	0.7435	0.113*	0.279 (5)
H24B	0.5265	0.2881	0.5994	0.113*	0.279 (5)
H24C	0.5293	0.2721	0.7726	0.113*	0.279 (5)
C242	0.7596 (19)	0.3538 (9)	0.7150 (19)	0.0628 (12)	0.279 (5)
H24D	0.8293	0.3241	0.7283	0.094*	0.279 (5)
H24E	0.8109	0.3904	0.7617	0.094*	0.279 (5)
H24F	0.6844	0.3584	0.5914	0.094*	0.279 (5)
C271	0.712 (2)	0.3858 (8)	1.251 (2)	0.0862 (18)	0.279 (5)
H27B	0.7862	0.3919	1.3738	0.129*	0.279 (5)
H27C	0.7005	0.3444	1.2248	0.129*	0.279 (5)
H27D	0.6184	0.4017	1.2255	0.129*	0.279 (5)
C272	0.8240 (16)	0.4773 (5)	1.214 (2)	0.0728 (14)	0.279 (5)
H27E	0.9107	0.4726	1.3308	0.109*	0.279 (5)
H27F	0.7512	0.5005	1.2199	0.109*	0.279 (5)
H27G	0.8514	0.4970	1.1395	0.109*	0.279 (5)
C31	0.2485 (3)	0.35915 (10)	0.0457 (3)	0.0502 (5)	
C32	0.3287 (4)	0.30983 (13)	0.0538 (5)	0.0740 (8)	
H32	0.3923	0.2917	0.1626	0.089*	
C33	0.3148 (6)	0.28750 (18)	−0.0981 (6)	0.1000 (13)	
H33	0.3683	0.2542	−0.0921	0.120*	
C34	0.2215 (6)	0.31457 (19)	−0.2594 (6)	0.0990 (13)	
H34	0.2133	0.2998	−0.3617	0.119*	
C35	0.1415 (5)	0.36271 (17)	−0.2695 (4)	0.0819 (10)	
H35	0.0779	0.3805	−0.3788	0.098*	
C36	0.1545 (3)	0.38549 (13)	−0.1169 (4)	0.0613 (6)	
H36	0.0998	0.4185	−0.1243	0.074*	
C37	0.2680 (3)	0.38409 (10)	0.2139 (3)	0.0494 (5)	
O31	0.3392 (3)	0.35292 (8)	0.3508 (3)	0.0804 (7)	
O32	0.2140 (2)	0.43361 (7)	0.2089 (3)	0.0545 (4)	

### 2-Amino-4,4,7,7-tetramethyl-4,5,6,7-tetrahydro-1,3-benzothiazol-3-ium benzoate (I) Atomic displacement parameters (Å^2^)

**Table:**

	*U*^11^	*U*^22^	*U*^33^	*U*^12^	*U*^13^	*U*^23^
S11	0.0441 (3)	0.0619 (8)	0.0344 (11)	0.0061 (4)	0.0196 (7)	−0.0073 (8)
C12	0.0386 (15)	0.0491 (16)	0.0332 (19)	−0.0017 (15)	0.0196 (14)	−0.0011 (18)
N13	0.0469 (9)	0.0461 (8)	0.0322 (10)	−0.0001 (7)	0.0206 (12)	−0.0033 (12)
C13A	0.0416 (16)	0.0388 (18)	0.0389 (10)	−0.0033 (15)	0.0238 (10)	0.0019 (9)
C14	0.0578 (16)	0.0395 (15)	0.0527 (14)	0.0071 (12)	0.0328 (12)	0.0066 (11)
C15	0.060 (2)	0.071 (3)	0.062 (2)	0.0243 (18)	0.0287 (17)	0.0097 (18)
C16	0.077 (3)	0.068 (3)	0.0466 (19)	0.023 (2)	0.0205 (16)	0.0172 (17)
C17	0.0462 (12)	0.0668 (14)	0.0373 (12)	0.0025 (10)	0.0157 (10)	0.0018 (10)
C17A	0.0409 (10)	0.0495 (11)	0.0370 (12)	0.0014 (9)	0.0202 (9)	0.0003 (9)
N12	0.0397 (18)	0.056 (3)	0.0379 (13)	0.002 (2)	0.0158 (12)	−0.0027 (15)
C141	0.101 (4)	0.0433 (15)	0.092 (5)	−0.001 (2)	0.058 (4)	−0.004 (3)
C142	0.072 (3)	0.0707 (17)	0.059 (3)	0.016 (2)	0.044 (3)	0.004 (3)
C171	0.075 (2)	0.147 (6)	0.032 (2)	−0.003 (4)	0.0249 (18)	−0.010 (3)
C172	0.048 (3)	0.077 (2)	0.062 (2)	−0.003 (2)	0.008 (2)	−0.0082 (17)
S21	0.0441 (3)	0.0619 (8)	0.0344 (11)	0.0061 (4)	0.0196 (7)	−0.0073 (8)
C22	0.0386 (15)	0.0491 (16)	0.0332 (19)	−0.0017 (15)	0.0196 (14)	−0.0011 (18)
N23	0.0469 (9)	0.0461 (8)	0.0322 (10)	−0.0001 (7)	0.0206 (12)	−0.0033 (12)
C23A	0.0416 (16)	0.0388 (18)	0.0389 (10)	−0.0033 (15)	0.0238 (10)	0.0019 (9)
C24	0.0578 (16)	0.0395 (15)	0.0527 (14)	0.0071 (12)	0.0328 (12)	0.0066 (11)
C25	0.060 (2)	0.071 (3)	0.062 (2)	0.0243 (18)	0.0287 (17)	0.0097 (18)
C26	0.077 (3)	0.068 (3)	0.0466 (19)	0.023 (2)	0.0205 (16)	0.0172 (17)
C27	0.0462 (12)	0.0668 (14)	0.0373 (12)	0.0025 (10)	0.0157 (10)	0.0018 (10)
C27A	0.0409 (10)	0.0495 (11)	0.0370 (12)	0.0014 (9)	0.0202 (9)	0.0003 (9)
N22	0.0397 (18)	0.056 (3)	0.0379 (13)	0.002 (2)	0.0158 (12)	−0.0027 (15)
C241	0.101 (4)	0.0433 (15)	0.092 (5)	−0.001 (2)	0.058 (4)	−0.004 (3)
C242	0.072 (3)	0.0707 (17)	0.059 (3)	0.016 (2)	0.044 (3)	0.004 (3)
C271	0.075 (2)	0.147 (6)	0.032 (2)	−0.003 (4)	0.0249 (18)	−0.010 (3)
C272	0.048 (3)	0.077 (2)	0.062 (2)	−0.003 (2)	0.008 (2)	−0.0082 (17)
C31	0.0518 (12)	0.0551 (12)	0.0448 (12)	−0.0198 (10)	0.0266 (10)	−0.0130 (9)
C32	0.090 (2)	0.0652 (16)	0.0702 (19)	−0.0049 (14)	0.0451 (17)	−0.0100 (13)
C33	0.127 (3)	0.089 (2)	0.102 (3)	−0.004 (2)	0.073 (3)	−0.031 (2)
C34	0.136 (3)	0.111 (3)	0.081 (3)	−0.032 (3)	0.078 (3)	−0.040 (2)
C35	0.091 (2)	0.109 (2)	0.0433 (15)	−0.0306 (19)	0.0335 (16)	−0.0161 (14)
C36	0.0566 (14)	0.0769 (16)	0.0412 (13)	−0.0154 (12)	0.0198 (11)	−0.0109 (11)
C37	0.0532 (12)	0.0525 (12)	0.0377 (11)	−0.0156 (10)	0.0211 (10)	−0.0090 (9)
O31	0.1175 (18)	0.0597 (10)	0.0402 (10)	0.0012 (10)	0.0260 (11)	−0.0053 (7)
O32	0.0614 (9)	0.0582 (9)	0.0454 (9)	−0.0052 (7)	0.0294 (8)	−0.0068 (7)

### 2-Amino-4,4,7,7-tetramethyl-4,5,6,7-tetrahydro-1,3-benzothiazol-3-ium benzoate (I) Geometric parameters (Å, º)

**Table:**

S11—C12	1.726 (3)	C23A—C24	1.506 (5)
S11—C17A	1.756 (3)	C24—C241	1.534 (7)
C12—N13	1.323 (3)	C24—C25	1.541 (7)
C12—N12	1.329 (6)	C24—C242	1.542 (7)
N13—C13A	1.396 (5)	C25—C26	1.513 (7)
N13—H13	0.8600	C25—H25A	0.9700
C13A—C17A	1.348 (4)	C25—H25B	0.9700
C13A—C14	1.504 (3)	C26—C27	1.535 (7)
C14—C141	1.535 (5)	C26—H26A	0.9700
C14—C142	1.542 (4)	C26—H26B	0.9700
C14—C15	1.543 (5)	C27—C27A	1.504 (5)
C15—C16	1.513 (5)	C27—C272	1.528 (7)
C15—H15A	0.9700	C27—C271	1.533 (7)
C15—H15B	0.9700	N22—H22A	0.8600
C16—C17	1.534 (5)	N22—H22B	0.8600
C16—H16A	0.9700	C241—H24A	0.9600
C16—H16B	0.9700	C241—H24B	0.9600
C17—C17A	1.505 (3)	C241—H24C	0.9600
C17—C172	1.530 (5)	C242—H24D	0.9600
C17—C171	1.534 (5)	C242—H24E	0.9600
N12—H12A	0.8600	C242—H24F	0.9600
N12—H12B	0.8600	C271—H27B	0.9600
C141—H14A	0.9600	C271—H27C	0.9600
C141—H14B	0.9600	C271—H27D	0.9600
C141—H14C	0.9600	C272—H27E	0.9600
C142—H14D	0.9600	C272—H27F	0.9600
C142—H14E	0.9600	C272—H27G	0.9600
C142—H14F	0.9600	C31—C36	1.381 (4)
C171—H17B	0.9600	C31—C32	1.385 (4)
C171—H17C	0.9600	C31—C37	1.508 (3)
C171—H17D	0.9600	C32—C33	1.376 (5)
C172—H17E	0.9600	C32—H32	0.9300
C172—H17F	0.9600	C33—C34	1.379 (7)
C172—H17G	0.9600	C33—H33	0.9300
S21—C22	1.726 (6)	C34—C35	1.357 (6)
S21—C27A	1.754 (5)	C34—H34	0.9300
C22—N23	1.323 (6)	C35—C36	1.389 (4)
C22—N22	1.328 (8)	C35—H35	0.9300
N23—C23A	1.399 (8)	C36—H36	0.9300
N23—H23	0.8600	C37—O32	1.252 (3)
C23A—C27A	1.350 (6)	C37—O31	1.255 (3)
			
C12—S11—C17A	90.29 (17)	C23A—C24—C25	107.5 (6)
N13—C12—N12	124.1 (4)	C241—C24—C25	113.1 (7)
N13—C12—S11	111.6 (2)	C23A—C24—C242	109.5 (7)
N12—C12—S11	124.3 (3)	C241—C24—C242	107.9 (7)
C12—N13—C13A	114.4 (3)	C25—C24—C242	109.3 (7)
C12—N13—H13	122.8	C26—C25—C24	114.4 (7)
C13A—N13—H13	122.8	C26—C25—H25A	108.7
C17A—C13A—N13	112.8 (3)	C24—C25—H25A	108.7
C17A—C13A—C14	125.1 (3)	C26—C25—H25B	108.7
N13—C13A—C14	121.8 (3)	C24—C25—H25B	108.7
C13A—C14—C141	109.7 (3)	H25A—C25—H25B	107.6
C13A—C14—C142	110.3 (3)	C25—C26—C27	113.8 (7)
C141—C14—C142	108.1 (3)	C25—C26—H26A	108.8
C13A—C14—C15	106.6 (3)	C27—C26—H26A	108.8
C141—C14—C15	112.9 (3)	C25—C26—H26B	108.8
C142—C14—C15	109.3 (3)	C27—C26—H26B	108.8
C16—C15—C14	114.4 (3)	H26A—C26—H26B	107.7
C16—C15—H15A	108.6	C27A—C27—C272	111.3 (7)
C14—C15—H15A	108.6	C27A—C27—C271	109.8 (7)
C16—C15—H15B	108.6	C272—C27—C271	109.8 (7)
C14—C15—H15B	108.6	C27A—C27—C26	105.5 (6)
H15A—C15—H15B	107.6	C272—C27—C26	110.5 (7)
C15—C16—C17	114.3 (3)	C271—C27—C26	109.8 (7)
C15—C16—H16A	108.7	C23A—C27A—C27	126.6 (6)
C17—C16—H16A	108.7	C23A—C27A—S21	110.4 (5)
C15—C16—H16B	108.7	C27—C27A—S21	122.7 (5)
C17—C16—H16B	108.7	C22—N22—H22A	120.0
H16A—C16—H16B	107.6	C22—N22—H22B	120.0
C17A—C17—C172	110.5 (3)	H22A—N22—H22B	120.0
C17A—C17—C171	109.5 (3)	C24—C241—H24A	109.5
C172—C17—C171	110.0 (4)	C24—C241—H24B	109.5
C17A—C17—C16	106.7 (3)	H24A—C241—H24B	109.5
C172—C17—C16	110.1 (4)	C24—C241—H24C	109.5
C171—C17—C16	109.9 (4)	H24A—C241—H24C	109.5
C13A—C17A—C17	127.4 (3)	H24B—C241—H24C	109.5
C13A—C17A—S11	110.5 (2)	C24—C242—H24D	109.5
C17—C17A—S11	122.1 (2)	C24—C242—H24E	109.5
C12—N12—H12A	120.0	H24D—C242—H24E	109.5
C12—N12—H12B	120.0	C24—C242—H24F	109.5
H12A—N12—H12B	120.0	H24D—C242—H24F	109.5
C14—C141—H14A	109.5	H24E—C242—H24F	109.5
C14—C141—H14B	109.5	C27—C271—H27B	109.5
H14A—C141—H14B	109.5	C27—C271—H27C	109.5
C14—C141—H14C	109.5	H27B—C271—H27C	109.5
H14A—C141—H14C	109.5	C27—C271—H27D	109.5
H14B—C141—H14C	109.5	H27B—C271—H27D	109.5
C14—C142—H14D	109.5	H27C—C271—H27D	109.5
C14—C142—H14E	109.5	C27—C272—H27E	109.5
H14D—C142—H14E	109.5	C27—C272—H27F	109.5
C14—C142—H14F	109.5	H27E—C272—H27F	109.5
H14D—C142—H14F	109.5	C27—C272—H27G	109.5
H14E—C142—H14F	109.5	H27E—C272—H27G	109.5
C17—C171—H17B	109.5	H27F—C272—H27G	109.5
C17—C171—H17C	109.5	C36—C31—C32	119.1 (2)
H17B—C171—H17C	109.5	C36—C31—C37	121.1 (2)
C17—C171—H17D	109.5	C32—C31—C37	119.8 (2)
H17B—C171—H17D	109.5	C33—C32—C31	120.4 (3)
H17C—C171—H17D	109.5	C33—C32—H32	119.8
C17—C172—H17E	109.5	C31—C32—H32	119.8
C17—C172—H17F	109.5	C32—C33—C34	119.9 (4)
H17E—C172—H17F	109.5	C32—C33—H33	120.0
C17—C172—H17G	109.5	C34—C33—H33	120.0
H17E—C172—H17G	109.5	C35—C34—C33	120.3 (3)
H17F—C172—H17G	109.5	C35—C34—H34	119.8
C22—S21—C27A	90.2 (4)	C33—C34—H34	119.8
N23—C22—N22	124.3 (10)	C34—C35—C36	120.2 (3)
N23—C22—S21	111.4 (6)	C34—C35—H35	119.9
N22—C22—S21	124.2 (9)	C36—C35—H35	119.9
C22—N23—C23A	113.8 (10)	C31—C36—C35	120.1 (3)
C22—N23—H23	123.1	C31—C36—H36	119.9
C23A—N23—H23	123.1	C35—C36—H36	119.9
C27A—C23A—N23	112.0 (8)	O32—C37—O31	124.7 (2)
C27A—C23A—C24	125.1 (6)	O32—C37—C31	119.0 (2)
N23—C23A—C24	120.8 (7)	O31—C37—C31	116.4 (2)
C23A—C24—C241	109.6 (7)		
			
C17A—S11—C12—N13	2 (2)	C27A—C23A—C24—C241	141 (2)
C17A—S11—C12—N12	−177 (3)	N23—C23A—C24—C241	−57 (4)
N12—C12—N13—C13A	174 (3)	C27A—C23A—C24—C25	17 (2)
S11—C12—N13—C13A	−5 (3)	N23—C23A—C24—C25	180 (4)
C12—N13—C13A—C17A	7 (3)	C27A—C23A—C24—C242	−101 (2)
C12—N13—C13A—C14	−178.9 (19)	N23—C23A—C24—C242	61 (4)
C17A—C13A—C14—C141	105.5 (8)	C23A—C24—C25—C26	−41.1 (12)
N13—C13A—C14—C141	−67.5 (15)	C241—C24—C25—C26	−162.2 (11)
C17A—C13A—C14—C142	−135.6 (8)	C242—C24—C25—C26	77.6 (12)
N13—C13A—C14—C142	51.5 (15)	C24—C25—C26—C27	61.4 (12)
C17A—C13A—C14—C15	−17.0 (8)	C25—C26—C27—C27A	−47.7 (11)
N13—C13A—C14—C15	170.0 (14)	C25—C26—C27—C272	−168.1 (10)
C13A—C14—C15—C16	44.4 (5)	C25—C26—C27—C271	70.6 (11)
C141—C14—C15—C16	−76.0 (5)	N23—C23A—C27A—C27	−175 (4)
C142—C14—C15—C16	163.6 (4)	C24—C23A—C27A—C27	−11 (3)
C14—C15—C16—C17	−61.3 (5)	N23—C23A—C27A—S21	12 (4)
C15—C16—C17—C17A	41.1 (6)	C24—C23A—C27A—S21	175.8 (17)
C15—C16—C17—C172	−78.9 (5)	C272—C27—C27A—C23A	144 (2)
C15—C16—C17—C171	159.7 (4)	C271—C27—C27A—C23A	−94 (2)
N13—C13A—C17A—C17	176.4 (14)	C26—C27—C27A—C23A	25 (2)
C14—C13A—C17A—C17	2.9 (13)	C272—C27—C27A—S21	−43 (2)
N13—C13A—C17A—S11	−5.7 (15)	C271—C27—C27A—S21	79 (2)
C14—C13A—C17A—S11	−179.2 (6)	C26—C27—C27A—S21	−163.0 (19)
C172—C17—C17A—C13A	105.8 (9)	C22—S21—C27A—C23A	−4 (4)
C171—C17—C17A—C13A	−132.9 (9)	C22—S21—C27A—C27	−178 (4)
C16—C17—C17A—C13A	−14.0 (9)	C36—C31—C32—C33	0.1 (4)
C172—C17—C17A—S11	−71.9 (8)	C37—C31—C32—C33	−178.4 (3)
C171—C17—C17A—S11	49.5 (8)	C31—C32—C33—C34	0.5 (6)
C16—C17—C17A—S11	168.4 (7)	C32—C33—C34—C35	−0.9 (6)
C12—S11—C17A—C13A	2.4 (15)	C33—C34—C35—C36	0.8 (6)
C12—S11—C17A—C17	−179.6 (14)	C32—C31—C36—C35	−0.2 (4)
C27A—S21—C22—N23	−5 (6)	C37—C31—C36—C35	178.2 (3)
C27A—S21—C22—N22	171 (8)	C34—C35—C36—C31	−0.2 (5)
N22—C22—N23—C23A	−162 (8)	C36—C31—C37—O32	−9.8 (3)
S21—C22—N23—C23A	13 (8)	C32—C31—C37—O32	168.6 (2)
C22—N23—C23A—C27A	−17 (7)	C36—C31—C37—O31	170.6 (2)
C22—N23—C23A—C24	179 (5)	C32—C31—C37—O31	−11.1 (3)

### 2-Amino-4,4,7,7-tetramethyl-4,5,6,7-tetrahydro-1,3-benzothiazol-3-ium benzoate (I) Hydrogen-bond geometry (Å, º)

**Table:**

*D*—H···*A*	*D*—H	H···*A*	*D*···*A*	*D*—H···*A*
N12—H12*A*···O32	0.86	2.10	2.918 (8)	158
N12—H12*B*···O32^i^	0.86	1.97	2.785 (9)	158
N13—H13···O31	0.86	1.77	2.621 (10)	174
N22—H22*A*···O32	0.86	2.13	2.86 (2)	142
N22—H22*B*···O32^i^	0.86	2.13	2.92 (2)	152
N23—H23···O31	0.86	1.74	2.56 (3)	157

Symmetry code: (i) *x*, −*y*+1, *z*+1/2.

### 2-Amino-4,4,7,7-tetramethyl-4,5,6,7-tetrahydro-1,3-benzothiazol-3-ium 2,4,6-trinitrophenolate (II) Crystal data

**Table:**

C_11_H_19_N_2_S^+^·C_6_H_2_N_3_O_7_^−^	*F*(000) = 920
*M**_r_* = 439.45	*D*_x_ = 1.399 Mg m^−^^3^
Monoclinic, *P*2_1_/*n*	Cu *K*α radiation, λ = 1.54178 Å
*a* = 10.7928 (2) Å	Cell parameters from 4122 reflections
*b* = 6.9591 (1) Å	θ = 4.6–72.4°
*c* = 28.0176 (5) Å	µ = 1.82 mm^−^^1^
β = 97.408 (1)°	*T* = 296 K
*V* = 2086.79 (6) Å^3^	Block, colourless
*Z* = 4	0.30 × 0.25 × 0.20 mm

### 2-Amino-4,4,7,7-tetramethyl-4,5,6,7-tetrahydro-1,3-benzothiazol-3-ium 2,4,6-trinitrophenolate (II) Data collection

**Table:**

Bruker Kappa APEXII diffractometer	3099 reflections with *I* > 2σ(*I*)
Radiation source: fine focus sealed tube	*R*_int_ = 0.060
φ and ω scans	θ_max_ = 72.4°, θ_min_ = 4.6°
Absorption correction: multi-scan (SADABS; Bruker, 2012)	*h* = −13→13
*T*_min_ = 0.696, *T*_max_ = 0.712	*k* = −7→8
40786 measured reflections	*l* = −34→34
4122 independent reflections	

### 2-Amino-4,4,7,7-tetramethyl-4,5,6,7-tetrahydro-1,3-benzothiazol-3-ium 2,4,6-trinitrophenolate (II) Refinement

**Table:**

Refinement on *F*^2^	46 restraints
Least-squares matrix: full	Hydrogen site location: inferred from neighbouring sites
*R*[*F*^2^ > 2σ(*F*^2^)] = 0.053	H-atom parameters constrained
*wR*(*F*^2^) = 0.139	*w* = 1/[σ^2^(*F*_o_^2^) + (0.0565*P*)^2^ + 0.8506*P*] where *P* = (*F*_o_^2^ + 2*F*_c_^2^)/3
*S* = 1.07	(Δ/σ)_max_ < 0.001
4122 reflections	Δρ_max_ = 0.25 e Å^−^^3^
336 parameters	Δρ_min_ = −0.23 e Å^−^^3^

### 2-Amino-4,4,7,7-tetramethyl-4,5,6,7-tetrahydro-1,3-benzothiazol-3-ium 2,4,6-trinitrophenolate (II) Special details

**Table:**

Geometry. All esds (except the esd in the dihedral angle between two l.s. planes) are estimated using the full covariance matrix. The cell esds are taken into account individually in the estimation of esds in distances, angles and torsion angles; correlations between esds in cell parameters are only used when they are defined by crystal symmetry. An approximate (isotropic) treatment of cell esds is used for estimating esds involving l.s. planes.

### 2-Amino-4,4,7,7-tetramethyl-4,5,6,7-tetrahydro-1,3-benzothiazol-3-ium 2,4,6-trinitrophenolate (II) Fractional atomic coordinates and isotropic or equivalent isotropic displacement parameters (Å^2^)

**Table:**

	*x*	*y*	*z*	*U*_iso_*/*U*_eq_	Occ. (<1)
S11	0.4474 (4)	0.4432 (9)	0.6202 (2)	0.0579 (9)	0.575 (4)
C12	0.4105 (9)	0.4371 (15)	0.5587 (3)	0.044 (2)	0.575 (4)
N13	0.313 (4)	0.550 (5)	0.5442 (3)	0.0520 (9)	0.575 (4)
H13	0.2818	0.5607	0.5145	0.062*	0.575 (4)
C13A	0.2642 (8)	0.6495 (11)	0.58137 (19)	0.0493 (12)	0.575 (4)
C14	0.1579 (5)	0.7881 (8)	0.57154 (15)	0.0599 (14)	0.575 (4)
C15	0.1124 (5)	0.8311 (9)	0.62028 (18)	0.0869 (13)	0.575 (4)
H15A	0.0592	0.9438	0.6168	0.104*	0.575 (4)
H15B	0.0621	0.7239	0.6287	0.104*	0.575 (4)
C16	0.2151 (14)	0.865 (2)	0.6605 (2)	0.091 (3)	0.575 (4)
H16A	0.1782	0.8970	0.6893	0.109*	0.575 (4)
H16B	0.2636	0.9748	0.6525	0.109*	0.575 (4)
C17	0.3043 (13)	0.6938 (18)	0.67201 (19)	0.0679 (9)	0.575 (4)
C17A	0.3331 (19)	0.618 (3)	0.6243 (2)	0.050 (2)	0.575 (4)
N12	0.4644 (8)	0.3246 (13)	0.5300 (3)	0.056 (2)	0.575 (4)
H12B	0.4370	0.3205	0.4998	0.067*	0.575 (4)
H12A	0.5272	0.2549	0.5413	0.067*	0.575 (4)
C141	0.2025 (7)	0.9702 (9)	0.5481 (2)	0.0885 (17)	0.575 (4)
H14A	0.2691	1.0281	0.5693	0.133*	0.575 (4)
H14B	0.1344	1.0593	0.5421	0.133*	0.575 (4)
H14C	0.2320	0.9373	0.5183	0.133*	0.575 (4)
C142	0.0507 (6)	0.6961 (13)	0.5378 (3)	0.094 (2)	0.575 (4)
H14D	0.0733	0.6893	0.5058	0.141*	0.575 (4)
H14E	−0.0234	0.7725	0.5376	0.141*	0.575 (4)
H14F	0.0352	0.5689	0.5489	0.141*	0.575 (4)
C171	0.4248 (14)	0.760 (3)	0.7026 (5)	0.105 (4)	0.575 (4)
H17B	0.4808	0.6526	0.7084	0.158*	0.575 (4)
H17C	0.4054	0.8091	0.7327	0.158*	0.575 (4)
H17D	0.4636	0.8585	0.6858	0.158*	0.575 (4)
C172	0.242 (2)	0.538 (2)	0.6987 (6)	0.105 (2)	0.575 (4)
H17E	0.1649	0.4996	0.6800	0.158*	0.575 (4)
H17F	0.2245	0.5866	0.7293	0.158*	0.575 (4)
H17G	0.2964	0.4287	0.7038	0.158*	0.575 (4)
S21	0.4188 (7)	0.4266 (12)	0.6178 (3)	0.0579 (9)	0.425 (4)
C22	0.3828 (15)	0.417 (2)	0.5563 (4)	0.044 (2)	0.425 (4)
N23	0.307 (5)	0.561 (7)	0.5400 (4)	0.0520 (9)	0.425 (4)
H23	0.2831	0.5796	0.5099	0.062*	0.425 (4)
C23A	0.2703 (12)	0.6809 (16)	0.5762 (3)	0.0493 (12)	0.425 (4)
C24	0.1832 (7)	0.8469 (10)	0.5648 (2)	0.0599 (14)	0.425 (4)
C25	0.1869 (7)	0.9644 (10)	0.6114 (2)	0.0869 (13)	0.425 (4)
H25A	0.2630	1.0403	0.6156	0.104*	0.425 (4)
H25B	0.1169	1.0530	0.6080	0.104*	0.425 (4)
C26	0.182 (2)	0.847 (3)	0.6557 (3)	0.091 (3)	0.425 (4)
H26A	0.1043	0.7751	0.6521	0.109*	0.425 (4)
H26B	0.1806	0.9331	0.6828	0.109*	0.425 (4)
C27	0.2907 (18)	0.705 (2)	0.6675 (2)	0.0679 (9)	0.425 (4)
C27A	0.312 (3)	0.614 (4)	0.6204 (3)	0.050 (2)	0.425 (4)
N22	0.4330 (12)	0.296 (2)	0.5284 (5)	0.056 (2)	0.425 (4)
H22B	0.4179	0.3075	0.4976	0.067*	0.425 (4)
H22A	0.4811	0.2060	0.5409	0.067*	0.425 (4)
C241	0.2272 (10)	0.9677 (13)	0.5243 (3)	0.0885 (17)	0.425 (4)
H24A	0.3150	0.9929	0.5316	0.133*	0.425 (4)
H24B	0.1823	1.0871	0.5215	0.133*	0.425 (4)
H24C	0.2116	0.8986	0.4945	0.133*	0.425 (4)
C242	0.0500 (8)	0.7745 (19)	0.5491 (4)	0.094 (2)	0.425 (4)
H24D	0.0485	0.6993	0.5203	0.141*	0.425 (4)
H24E	−0.0053	0.8823	0.5430	0.141*	0.425 (4)
H24F	0.0234	0.6967	0.5742	0.141*	0.425 (4)
C271	0.409 (2)	0.811 (4)	0.6896 (7)	0.105 (4)	0.425 (4)
H27B	0.4790	0.7257	0.6915	0.158*	0.425 (4)
H27C	0.3980	0.8546	0.7213	0.158*	0.425 (4)
H27D	0.4231	0.9196	0.6699	0.158*	0.425 (4)
C272	0.257 (3)	0.551 (3)	0.7023 (8)	0.105 (2)	0.425 (4)
H27E	0.1901	0.4730	0.6867	0.158*	0.425 (4)
H27F	0.2307	0.6109	0.7301	0.158*	0.425 (4)
H27G	0.3285	0.4714	0.7119	0.158*	0.425 (4)
C31	0.25836 (19)	0.3704 (3)	0.40355 (8)	0.0532 (5)	
O31	0.29348 (16)	0.4112 (3)	0.44628 (6)	0.0766 (5)	
C32	0.29371 (19)	0.1972 (3)	0.38012 (7)	0.0530 (5)	
N32	0.3787 (2)	0.0623 (3)	0.40611 (7)	0.0700 (6)	0.769 (7)
O32	0.4480 (4)	0.1078 (5)	0.44228 (11)	0.0910 (13)	0.769 (7)
O33	0.3829 (5)	−0.1031 (5)	0.38936 (14)	0.1098 (17)	0.769 (7)
N42	0.3787 (2)	0.0623 (3)	0.40611 (7)	0.0700 (6)	0.231 (7)
O42	0.3856 (13)	0.064 (2)	0.44958 (17)	0.0910 (13)	0.231 (7)
O43	0.4464 (12)	−0.036 (2)	0.3836 (4)	0.1098 (17)	0.231 (7)
C33	0.2505 (2)	0.1476 (4)	0.33364 (8)	0.0582 (6)	
H33	0.2754	0.0329	0.3208	0.070*	
C34	0.1701 (2)	0.2685 (4)	0.30623 (8)	0.0596 (6)	
N34	0.1239 (2)	0.2151 (4)	0.25722 (8)	0.0872 (8)	
O34	0.1630 (3)	0.0696 (4)	0.24086 (8)	0.1311 (11)	
O35	0.0470 (3)	0.3175 (4)	0.23433 (8)	0.1305 (10)	
C35	0.1340 (2)	0.4400 (4)	0.32450 (8)	0.0621 (6)	
H35	0.0815	0.5228	0.3053	0.075*	
C36	0.1758 (2)	0.4882 (4)	0.37107 (8)	0.0588 (6)	
N36	0.1302 (3)	0.6705 (4)	0.38765 (10)	0.0904 (7)	0.789 (6)
O36	0.0776 (4)	0.7824 (5)	0.35711 (13)	0.1188 (15)	0.789 (6)
O37	0.1469 (6)	0.7149 (7)	0.42959 (12)	0.153 (2)	0.789 (6)
N46	0.1302 (3)	0.6705 (4)	0.38765 (10)	0.0904 (7)	0.211 (6)
O46	0.0179 (6)	0.700 (2)	0.3785 (6)	0.1188 (15)	0.211 (6)
O47	0.2019 (12)	0.747 (3)	0.4177 (5)	0.153 (2)	0.211 (6)

### 2-Amino-4,4,7,7-tetramethyl-4,5,6,7-tetrahydro-1,3-benzothiazol-3-ium 2,4,6-trinitrophenolate (II) Atomic displacement parameters (Å^2^)

**Table:**

	*U*^11^	*U*^22^	*U*^33^	*U*^12^	*U*^13^	*U*^23^
S11	0.066 (2)	0.0644 (10)	0.0416 (6)	0.0050 (15)	0.0009 (14)	0.0011 (6)
C12	0.043 (5)	0.044 (2)	0.0440 (13)	−0.009 (3)	0.0029 (17)	−0.0006 (12)
N13	0.061 (3)	0.055 (4)	0.0391 (13)	0.0043 (12)	0.003 (3)	0.001 (3)
C13A	0.0550 (14)	0.047 (3)	0.0477 (15)	−0.0061 (19)	0.0127 (13)	0.0011 (15)
C14	0.060 (3)	0.061 (3)	0.060 (2)	0.006 (2)	0.0128 (16)	0.006 (2)
C15	0.086 (3)	0.097 (4)	0.080 (3)	0.031 (2)	0.020 (2)	−0.009 (3)
C16	0.098 (8)	0.108 (4)	0.071 (2)	0.019 (5)	0.021 (3)	−0.025 (2)
C17	0.068 (3)	0.090 (2)	0.0472 (15)	−0.0022 (16)	0.0108 (16)	−0.0111 (16)
C17A	0.048 (6)	0.0579 (15)	0.0447 (14)	−0.007 (3)	0.0052 (17)	−0.0053 (16)
N12	0.064 (5)	0.056 (3)	0.0463 (11)	0.007 (4)	0.002 (2)	−0.0030 (14)
C141	0.109 (4)	0.064 (2)	0.093 (5)	0.017 (2)	0.014 (4)	0.019 (4)
C142	0.0641 (18)	0.128 (8)	0.087 (4)	0.005 (3)	0.001 (2)	−0.001 (4)
C171	0.090 (4)	0.160 (11)	0.064 (8)	−0.009 (5)	−0.001 (4)	−0.050 (7)
C172	0.127 (5)	0.137 (4)	0.058 (2)	0.000 (4)	0.034 (3)	0.012 (3)
S21	0.066 (2)	0.0644 (10)	0.0416 (6)	0.0050 (15)	0.0009 (14)	0.0011 (6)
C22	0.043 (5)	0.044 (2)	0.0440 (13)	−0.009 (3)	0.0029 (17)	−0.0006 (12)
N23	0.061 (3)	0.055 (4)	0.0391 (13)	0.0043 (12)	0.003 (3)	0.001 (3)
C23A	0.0550 (14)	0.047 (3)	0.0477 (15)	−0.0061 (19)	0.0127 (13)	0.0011 (15)
C24	0.060 (3)	0.061 (3)	0.060 (2)	0.006 (2)	0.0128 (16)	0.006 (2)
C25	0.086 (3)	0.097 (4)	0.080 (3)	0.031 (2)	0.020 (2)	−0.009 (3)
C26	0.098 (8)	0.108 (4)	0.071 (2)	0.019 (5)	0.021 (3)	−0.025 (2)
C27	0.068 (3)	0.090 (2)	0.0472 (15)	−0.0022 (16)	0.0108 (16)	−0.0111 (16)
C27A	0.048 (6)	0.0579 (15)	0.0447 (14)	−0.007 (3)	0.0052 (17)	−0.0053 (16)
N22	0.064 (5)	0.056 (3)	0.0463 (11)	0.007 (4)	0.002 (2)	−0.0030 (14)
C241	0.109 (4)	0.064 (2)	0.093 (5)	0.017 (2)	0.014 (4)	0.019 (4)
C242	0.0641 (18)	0.128 (8)	0.087 (4)	0.005 (3)	0.001 (2)	−0.001 (4)
C271	0.090 (4)	0.160 (11)	0.064 (8)	−0.009 (5)	−0.001 (4)	−0.050 (7)
C272	0.127 (5)	0.137 (4)	0.058 (2)	0.000 (4)	0.034 (3)	0.012 (3)
C31	0.0485 (11)	0.0600 (14)	0.0512 (12)	0.0002 (10)	0.0077 (9)	−0.0020 (10)
O31	0.0806 (11)	0.0887 (14)	0.0561 (10)	0.0218 (10)	−0.0078 (8)	−0.0209 (9)
C32	0.0527 (12)	0.0600 (14)	0.0462 (11)	0.0069 (10)	0.0059 (9)	0.0037 (10)
N32	0.0817 (14)	0.0742 (15)	0.0524 (11)	0.0250 (12)	0.0026 (10)	−0.0040 (10)
O32	0.089 (3)	0.111 (2)	0.0640 (14)	0.044 (2)	−0.0235 (16)	−0.0217 (14)
O33	0.137 (4)	0.074 (2)	0.105 (2)	0.040 (2)	−0.040 (2)	−0.0179 (19)
N42	0.0817 (14)	0.0742 (15)	0.0524 (11)	0.0250 (12)	0.0026 (10)	−0.0040 (10)
O42	0.089 (3)	0.111 (2)	0.0640 (14)	0.044 (2)	−0.0235 (16)	−0.0217 (14)
O43	0.137 (4)	0.074 (2)	0.105 (2)	0.040 (2)	−0.040 (2)	−0.0179 (19)
C33	0.0641 (13)	0.0621 (14)	0.0490 (12)	0.0062 (11)	0.0094 (10)	−0.0037 (11)
C34	0.0591 (13)	0.0759 (17)	0.0435 (11)	0.0057 (12)	0.0055 (9)	0.0009 (11)
N34	0.0957 (17)	0.110 (2)	0.0518 (12)	0.0274 (16)	−0.0050 (11)	−0.0053 (13)
O34	0.157 (2)	0.158 (3)	0.0699 (14)	0.065 (2)	−0.0203 (14)	−0.0428 (15)
O35	0.155 (2)	0.150 (2)	0.0721 (13)	0.059 (2)	−0.0394 (14)	−0.0070 (14)
C35	0.0589 (13)	0.0703 (16)	0.0569 (13)	0.0103 (12)	0.0069 (10)	0.0099 (12)
C36	0.0602 (13)	0.0574 (14)	0.0602 (13)	0.0076 (11)	0.0128 (11)	−0.0016 (11)
N36	0.1061 (19)	0.0778 (18)	0.0864 (18)	0.0249 (15)	0.0094 (14)	−0.0097 (15)
O36	0.138 (3)	0.086 (2)	0.125 (3)	0.054 (2)	−0.010 (2)	−0.0002 (19)
O37	0.279 (5)	0.112 (3)	0.072 (2)	0.085 (3)	0.034 (2)	−0.014 (2)
N46	0.1061 (19)	0.0778 (18)	0.0864 (18)	0.0249 (15)	0.0094 (14)	−0.0097 (15)
O46	0.138 (3)	0.086 (2)	0.125 (3)	0.054 (2)	−0.010 (2)	−0.0002 (19)
O47	0.279 (5)	0.112 (3)	0.072 (2)	0.085 (3)	0.034 (2)	−0.014 (2)

### 2-Amino-4,4,7,7-tetramethyl-4,5,6,7-tetrahydro-1,3-benzothiazol-3-ium 2,4,6-trinitrophenolate (II) Geometric parameters (Å, º)

**Table:**

S11—C12	1.719 (3)	C24—C241	1.535 (6)
S11—C17A	1.746 (3)	C24—C25	1.538 (6)
C12—N12	1.312 (3)	C25—C26	1.493 (13)
C12—N13	1.336 (9)	C25—H25A	0.9700
N13—C13A	1.407 (5)	C25—H25B	0.9700
N13—H13	0.8600	C26—C27	1.537 (6)
C13A—C17A	1.347 (6)	C26—H26A	0.9700
C13A—C14	1.498 (4)	C26—H26B	0.9700
C14—C142	1.536 (5)	C27—C27A	1.507 (4)
C14—C141	1.533 (6)	C27—C272	1.526 (5)
C14—C15	1.538 (5)	C27—C271	1.533 (5)
C15—C16	1.494 (12)	N22—H22B	0.8600
C15—H15A	0.9700	N22—H22A	0.8600
C15—H15B	0.9700	C241—H24A	0.9600
C16—C17	1.538 (5)	C241—H24B	0.9600
C16—H16A	0.9700	C241—H24C	0.9600
C16—H16B	0.9700	C242—H24D	0.9600
C17—C17A	1.507 (4)	C242—H24E	0.9600
C17—C172	1.526 (5)	C242—H24F	0.9600
C17—C171	1.532 (5)	C271—H27B	0.9600
N12—H12B	0.8600	C271—H27C	0.9600
N12—H12A	0.8600	C271—H27D	0.9600
C141—H14A	0.9600	C272—H27E	0.9600
C141—H14B	0.9600	C272—H27F	0.9600
C141—H14C	0.9600	C272—H27G	0.9600
C142—H14D	0.9600	C31—O31	1.241 (3)
C142—H14E	0.9600	C31—C36	1.444 (3)
C142—H14F	0.9600	C31—C32	1.447 (3)
C171—H17B	0.9600	C32—C33	1.370 (3)
C171—H17C	0.9600	C32—N32	1.442 (3)
C171—H17D	0.9600	N32—O32	1.221 (3)
C172—H17E	0.9600	N32—O33	1.246 (3)
C172—H17F	0.9600	C33—C34	1.370 (3)
C172—H17G	0.9600	C33—H33	0.9300
S21—C22	1.719 (4)	C34—C35	1.375 (3)
S21—C27A	1.746 (4)	C34—N34	1.448 (3)
C22—N22	1.311 (4)	N34—O34	1.209 (3)
C22—N23	1.336 (9)	N34—O35	1.213 (3)
N23—C23A	1.407 (6)	C35—C36	1.366 (3)
N23—H23	0.8600	C35—H35	0.9300
C23A—C27A	1.347 (6)	C36—N36	1.458 (3)
C23A—C24	1.497 (5)	N36—O37	1.206 (4)
C24—C242	1.534 (6)	N36—O36	1.239 (3)
			
C12—S11—C17A	90.46 (18)	C242—C24—C25	109.8 (5)
N12—C12—N13	124.0 (4)	C241—C24—C25	111.1 (5)
N12—C12—S11	124.5 (3)	C26—C25—C24	114.5 (7)
N13—C12—S11	111.2 (3)	C26—C25—H25A	108.6
C12—N13—C13A	114.8 (4)	C24—C25—H25A	108.6
C12—N13—H13	122.6	C26—C25—H25B	108.6
C13A—N13—H13	122.6	C24—C25—H25B	108.6
C17A—C13A—N13	111.4 (6)	H25A—C25—H25B	107.6
C17A—C13A—C14	126.2 (3)	C25—C26—C27	114.9 (9)
N13—C13A—C14	122.0 (4)	C25—C26—H26A	108.5
C13A—C14—C142	110.0 (4)	C27—C26—H26A	108.5
C13A—C14—C141	109.6 (4)	C25—C26—H26B	108.5
C142—C14—C141	109.6 (4)	C27—C26—H26B	108.5
C13A—C14—C15	106.6 (3)	H26A—C26—H26B	107.5
C142—C14—C15	109.0 (4)	C27A—C27—C272	109.8 (5)
C141—C14—C15	111.9 (4)	C27A—C27—C271	110.1 (5)
C16—C15—C14	114.2 (5)	C272—C27—C271	109.6 (5)
C16—C15—H15A	108.7	C27A—C27—C26	106.4 (4)
C14—C15—H15A	108.7	C272—C27—C26	110.5 (5)
C16—C15—H15B	108.7	C271—C27—C26	110.4 (5)
C14—C15—H15B	108.7	C23A—C27A—C27	126.0 (5)
H15A—C15—H15B	107.6	C23A—C27A—S21	111.4 (4)
C15—C16—C17	114.8 (8)	C27—C27A—S21	121.7 (3)
C15—C16—H16A	108.6	C22—N22—H22B	120.0
C17—C16—H16A	108.6	C22—N22—H22A	120.0
C15—C16—H16B	108.6	H22B—N22—H22A	120.0
C17—C16—H16B	108.6	C24—C241—H24A	109.5
H16A—C16—H16B	107.5	C24—C241—H24B	109.5
C17A—C17—C172	109.9 (4)	H24A—C241—H24B	109.5
C17A—C17—C171	110.1 (4)	C24—C241—H24C	109.5
C172—C17—C171	109.6 (4)	H24A—C241—H24C	109.5
C17A—C17—C16	106.3 (3)	H24B—C241—H24C	109.5
C172—C17—C16	110.4 (4)	C24—C242—H24D	109.5
C171—C17—C16	110.4 (4)	C24—C242—H24E	109.5
C13A—C17A—C17	125.6 (5)	H24D—C242—H24E	109.5
C13A—C17A—S11	111.6 (3)	C24—C242—H24F	109.5
C17—C17A—S11	122.0 (3)	H24D—C242—H24F	109.5
C12—N12—H12B	120.0	H24E—C242—H24F	109.5
C12—N12—H12A	120.0	C27—C271—H27B	109.5
H12B—N12—H12A	120.0	C27—C271—H27C	109.5
C14—C141—H14A	109.5	H27B—C271—H27C	109.5
C14—C141—H14B	109.5	C27—C271—H27D	109.5
H14A—C141—H14B	109.5	H27B—C271—H27D	109.5
C14—C141—H14C	109.5	H27C—C271—H27D	109.5
H14A—C141—H14C	109.5	C27—C272—H27E	109.5
H14B—C141—H14C	109.5	C27—C272—H27F	109.5
C14—C142—H14D	109.5	H27E—C272—H27F	109.5
C14—C142—H14E	109.5	C27—C272—H27G	109.5
H14D—C142—H14E	109.5	H27E—C272—H27G	109.5
C14—C142—H14F	109.5	H27F—C272—H27G	109.5
H14D—C142—H14F	109.5	O31—C31—C36	124.4 (2)
H14E—C142—H14F	109.5	O31—C31—C32	124.2 (2)
C17—C171—H17B	109.5	C36—C31—C32	111.38 (19)
C17—C171—H17C	109.5	C33—C32—N32	115.6 (2)
H17B—C171—H17C	109.5	C33—C32—C31	124.3 (2)
C17—C171—H17D	109.5	N32—C32—C31	120.05 (19)
H17B—C171—H17D	109.5	O32—N32—O33	120.2 (3)
H17C—C171—H17D	109.5	O32—N32—C32	122.1 (3)
C17—C172—H17E	109.5	O33—N32—C32	117.6 (2)
C17—C172—H17F	109.5	C32—C33—C34	119.4 (2)
H17E—C172—H17F	109.5	C32—C33—H33	120.3
C17—C172—H17G	109.5	C34—C33—H33	120.3
H17E—C172—H17G	109.5	C33—C34—C35	120.9 (2)
H17F—C172—H17G	109.5	C33—C34—N34	119.3 (2)
C22—S21—C27A	90.3 (2)	C35—C34—N34	119.8 (2)
N22—C22—N23	124.0 (5)	O34—N34—O35	122.7 (3)
N22—C22—S21	124.5 (5)	O34—N34—C34	118.7 (2)
N23—C22—S21	111.2 (4)	O35—N34—C34	118.6 (3)
C22—N23—C23A	114.6 (4)	C36—C35—C34	119.6 (2)
C22—N23—H23	122.7	C36—C35—H35	120.2
C23A—N23—H23	122.7	C34—C35—H35	120.2
C27A—C23A—N23	111.4 (7)	C35—C36—C31	124.3 (2)
C27A—C23A—C24	126.1 (4)	C35—C36—N36	115.7 (2)
N23—C23A—C24	122.1 (4)	C31—C36—N36	120.0 (2)
C23A—C24—C242	110.3 (5)	O37—N36—O36	120.6 (3)
C23A—C24—C241	109.5 (5)	O37—N36—C36	121.3 (3)
C242—C24—C241	109.6 (5)	O36—N36—C36	118.0 (3)
C23A—C24—C25	106.4 (4)		
			
C17A—S11—C12—N12	−179.1 (15)	C241—C24—C25—C26	−163.1 (10)
C17A—S11—C12—N13	−4 (3)	C24—C25—C26—C27	61.0 (16)
N12—C12—N13—C13A	176 (2)	C25—C26—C27—C27A	−42.5 (15)
S11—C12—N13—C13A	1 (4)	C25—C26—C27—C272	−161.7 (14)
C12—N13—C13A—C17A	4 (5)	C25—C26—C27—C271	76.9 (15)
C12—N13—C13A—C14	178 (2)	N23—C23A—C27A—C27	180 (4)
C17A—C13A—C14—C142	−138.0 (17)	C24—C23A—C27A—C27	−7 (4)
N13—C13A—C14—C142	49 (3)	N23—C23A—C27A—S21	10 (4)
C17A—C13A—C14—C141	101.4 (17)	C24—C23A—C27A—S21	−176.7 (13)
N13—C13A—C14—C141	−71 (3)	C272—C27—C27A—C23A	137 (3)
C17A—C13A—C14—C15	−19.9 (18)	C271—C27—C27A—C23A	−103 (3)
N13—C13A—C14—C15	167 (3)	C26—C27—C27A—C23A	17 (3)
C13A—C14—C15—C16	43.7 (9)	C272—C27—C27A—S21	−55 (3)
C142—C14—C15—C16	162.5 (8)	C271—C27—C27A—S21	66 (3)
C141—C14—C15—C16	−76.1 (9)	C26—C27—C27A—S21	−174 (3)
C14—C15—C16—C17	−61.2 (12)	C22—S21—C27A—C23A	−10 (3)
C15—C16—C17—C17A	44.2 (11)	C22—S21—C27A—C27	180 (2)
C15—C16—C17—C172	−75.0 (11)	O31—C31—C32—C33	176.8 (2)
C15—C16—C17—C171	163.6 (10)	C36—C31—C32—C33	−2.4 (3)
N13—C13A—C17A—C17	−177 (3)	O31—C31—C32—N32	−2.4 (4)
C14—C13A—C17A—C17	10 (3)	C36—C31—C32—N32	178.3 (2)
N13—C13A—C17A—S11	−7 (3)	C33—C32—N32—O32	160.8 (4)
C14—C13A—C17A—S11	179.6 (9)	C31—C32—N32—O32	−19.9 (4)
C172—C17—C17A—C13A	99.6 (19)	C33—C32—N32—O33	−16.4 (5)
C171—C17—C17A—C13A	−139.5 (18)	C31—C32—N32—O33	162.8 (4)
C16—C17—C17A—C13A	−19.9 (19)	N32—C32—C33—C34	−179.5 (2)
C172—C17—C17A—S11	−69.1 (18)	C31—C32—C33—C34	1.3 (4)
C171—C17—C17A—S11	51.7 (19)	C32—C33—C34—C35	1.1 (4)
C16—C17—C17A—S11	171.3 (18)	C32—C33—C34—N34	−179.6 (2)
C12—S11—C17A—C13A	6.6 (18)	C33—C34—N34—O34	−4.0 (4)
C12—S11—C17A—C17	176.7 (14)	C35—C34—N34—O34	175.3 (3)
C27A—S21—C22—N22	−180 (2)	C33—C34—N34—O35	175.6 (3)
C27A—S21—C22—N23	7 (4)	C35—C34—N34—O35	−5.1 (4)
N22—C22—N23—C23A	−176 (3)	C33—C34—C35—C36	−2.0 (4)
S21—C22—N23—C23A	−3 (6)	N34—C34—C35—C36	178.7 (2)
C22—N23—C23A—C27A	−5 (6)	C34—C35—C36—C31	0.6 (4)
C22—N23—C23A—C24	−178 (3)	C34—C35—C36—N36	−178.5 (2)
C27A—C23A—C24—C242	−100 (2)	O31—C31—C36—C35	−177.8 (2)
N23—C23A—C24—C242	72 (4)	C32—C31—C36—C35	1.5 (3)
C27A—C23A—C24—C241	139 (2)	O31—C31—C36—N36	1.3 (4)
N23—C23A—C24—C241	−48 (4)	C32—C31—C36—N36	−179.4 (2)
C27A—C23A—C24—C25	19 (2)	C35—C36—N36—O37	169.7 (4)
N23—C23A—C24—C25	−169 (4)	C31—C36—N36—O37	−9.4 (5)
C23A—C24—C25—C26	−43.9 (11)	C35—C36—N36—O36	−12.9 (5)
C242—C24—C25—C26	75.4 (11)	C31—C36—N36—O36	167.9 (3)

### 2-Amino-4,4,7,7-tetramethyl-4,5,6,7-tetrahydro-1,3-benzothiazol-3-ium 2,4,6-trinitrophenolate (II) Hydrogen-bond geometry (Å, º)

**Table:**

*D*—H···*A*	*D*—H	H···*A*	*D*···*A*	*D*—H···*A*
N12—H12*A*···O32^i^	0.86	2.57	3.219 (10)	133
N12—H12*A*···O33^i^	0.86	2.31	3.039 (10)	142
N12—H12*A*···O42^i^	0.86	2.41	3.166 (16)	147
N12—H12*A*···O43^i^	0.86	2.58	3.197 (15)	129
N22—H22*A*···O32^i^	0.86	2.34	3.154 (14)	158
N22—H22*A*···O33^i^	0.86	2.40	3.143 (14)	146
N22—H22*A*···O42^i^	0.86	2.36	3.190 (19)	163
N22—H22*A*···O43^i^	0.86	2.46	3.197 (18)	144
N12—H12*B*···O31	0.86	2.11	2.855 (9)	145
N12—H12*B*···O32	0.86	2.20	2.870 (9)	134
N12—H12*B*···O42	0.86	2.30	2.932 (13)	131
N22—H22*B*···O31	0.86	1.97	2.704 (14)	142
N22—H22*B*···O32	0.86	2.14	2.768 (14)	130
N22—H22*B*···O42	0.86	2.16	2.730 (17)	123
N13—H13···O31	0.86	2.19	2.891 (14)	138
N23—H23···O31	0.86	2.15	2.81 (2)	134

Symmetry code: (i) −*x*+1, −*y*, −*z*+1.
